# Evaluation of the Content of Micro- and Macroelements in Raspberries Depending on the Species, Cultivar Variety, and Geographical Environment

**DOI:** 10.3390/nu15173782

**Published:** 2023-08-30

**Authors:** Natalia Adamczuk, Justyna Ośko, Małgorzata Grembecka, Paweł Konieczyński, Piotr Migas, Agnieszka Orzeł, Barbara Baj-Wójtowicz, Mirosława Krauze-Baranowska

**Affiliations:** 1 Department of Pharmacognosy with Medicinal Plant Garden, Faculty of Pharmacy, Medical University of Gdansk, Al. Gen. J. Hallera 107, 80-416 Gdańsk, Poland; natalia.adamczuk@gumed.edu.pl (N.A.); piotr.migas@gumed.edu.pl (P.M.); 2 Department of Bromatology, Faculty of Pharmacy, Medical University of Gdansk, Al. Gen. J. Hallera 107, 80-416 Gdańsk, Poland; justyna.osko@gumed.edu.pl (J.O.); malgorzata.grembecka@gumed.edu.pl (M.G.); 3 Department of Analytical Chemistry, Faculty of Pharmacy, Medical University of Gdansk, Al. Gen. J. Hallera 107, 80-416 Gdańsk, Poland; pawel.konieczynski@gumed.edu.pl; 4 Niwa Berry Breeding Company, Brzezna 1, 33-386 Podegrodzie, Poland; agnieszka.orzel@niwabrzezna.pl; 5 Taylor Institute, University of Oxford, St. Giles, Oxford OX1 2JD, UK; biuro@lubelskieziola.pl

**Keywords:** *Rubus*, raspberry fruits, macroelements, microelements, chemometric analysis

## Abstract

The study aimed to analyse the macro- and micro-nutrient content in fruits of *Rubus* species (*R. idaeus*, *R. occidentalis*, *R. chamaemorus*, and *R. chingii*) and their varieties or hybrids from different regions. Flame atomic absorption spectrometry with deuterium background correction was used to measure concentrations of nine essential elements (K, Mg, Ca, Na, Mn, Fe, Cr, Zn, and Cu) and two heavy metals (Pb, Cd). Chemometric analysis compared the elemental profiles. Results confirmed raspberries as a rich source of macroelements (K, Mg) and microelements (Zn, Cu, Mn, Cr). The ‘Bristol’ cultivar consistently had the highest Fe content regardless of origin. Cr presence was observed in black raspberries for the first time. Previously observed relationships like K-Na antagonism and Cr/Zn, Fe/Zn synergism were found in raspberry fruits. Factor and cluster analyses demonstrated species and geographical diversity among Polish raspberry samples and clear separation of *R. chingii* from China. Raspberry fruits, due to the rich complex of polyphenols, are classified as superfoods, and the content of bioelements determined in them guarantees coverage of the daily requirement for macro- and microelements (RDA depending on the element: 5.6–204% for *R. idaeus*, 8.8–469, 4% for *R. occidentalis*, and 1.4–67.2% for *R. chamaemorus*), finally confirming this opinion.

## 1. Introduction

Fruits are considered “nutrient-rich foods” because they provide significant amounts of minerals and vitamins and are relatively low in calories [1]. One of the most popular berries in the world is red raspberry (*Rubus idaeus* L., *Rosaceae*), with an annual global production of about 168,000 tons [2]. Poland ranks third in the production of red raspberries in Europe and fourth in the world [3]. It is noteworthy that the species *Rubus idaeus*, belonging to the genus *Rubus* L., produces not only red fruits rich in anthocyanins but also some varieties of this species have yellow fruits and are anthocyanin free (e.g., ‘Poranna Rosa’ and ‘Promyk’) [4]. *Rubus* is one of the largest genera in the *Rosaceae* family and includes species that provide different fruits for direct consumption or processing in numerous industries, mainly the food and pharmaceutical sectors [5]. In contrast to the red and yellow fruits of *R. idaeus*, very popular in Eastern and Western Europe, the characteristic and dominant fruit in Scandinavian countries is the yellow cloudberry (*Rubus chamaemorus* L., *Rosaceae*) [6]. In Poland, this genus is under strict species protection, while in recent years, its fruits have been obtained with high efficiency from experimental crops [7]. In European folk medicine, cloudberries and red raspberries were used to treat respiratory tract diseases and colds [8]. The fruits of the *Rubus* L. genus, which are gaining importance in Poland, are the fruits of the black raspberry (*Rubus occidentalis* L. *Rosaceae*) and are classified as a so-called “superfood” [9]. Compared to other raspberries, black raspberries are characterized by a much higher content of anthocyanins and, depending on the variety, it can be almost four times higher [10]. *Rubus occidentalis* L. (black raspberry) is native to North America, mainly in the eastern US, where many cultivars are grown, the most popular of which are ‘Bristol’, ‘Jewel’, and ‘MacBlack’ [11]. Numerous varieties of black raspberry are also grown in Poland (e.g., ‘Heban’, ‘Litacz’, and ‘Niwot’), and the fruits obtained from them are becoming more and more popular due to their health-promoting properties [12]. In turn, *Rubus chingii* L. (*Rosaceae*) comes from China, and its fruits have been used for centuries in folk medicine as a weight loss aid and in the treatment of kidney and eye diseases [13].

Recently, the interest in raspberry fruit has been gradually increasing. Their antioxidant [14,15,16], anti-inflammatory [17,18], antibacterial [8], and anticancer [19] properties were confirmed in numerous in vitro [20,21,22,23,24], in vivo [25,26,27,28,29,30,31], and clinical studies [32,33,34,35], are associated with the presence of a number of groups of polyphenolic compounds (anthocyanins, ellagitannins, flavonols, and flavan-3-ols) and simple phenols (phenolic acids) [8,19,36,37]. In particular, these studies focus on red raspberry and black raspberry fruits, supporting the former’s use as anti-inflammatory and antimicrobial in the treatment of colds, which was previously practiced in folk medicine [38], and revealing their new application possibilities, e.g., in the treatment of colorectal cancer [23,39,40,41], gastric ulcer [31], rheumatoid arthritis [29,30], cardiovascular [42] and Alzheimer diseases [42,43], type 2 diabetes and metabolic syndrome [44,45].

In addition to many of the above-mentioned groups of biologically active secondary metabolites, raspberry fruits also contain other components, such as bio-elements, which play an important role in maintaining the homeostasis of the human body and its health [46,47]. Red and black raspberries are a rich source of macronutrients, including potassium (K) and calcium (Ca), magnesium (Mg), and zinc (Zn). So far, the content of basic bioelements (Na, K, Ca, Mg, Zn, Cu, Mn, and Fe) has been determined in some raspberry fruits—mainly red [48,49] and black [50,51], as well as cloudberries [52]. Moreover, the micronutrients such as copper (Cu) and iron (Fe) are present in higher concentrations in black raspberry fruits, and these fruits may play a significant role in the prevention of anemia [53]. On the other hand, the content of chromium (Cr) was determined only in fresh fruits of *R. idaeus* [54] and *R. chamaemorus* [52].

The assessment of the composition of macro- and microelements in the fruits of various species and varieties of raspberries in relation to the recommended intake (RDA) for an adult is important information not only for dieticians, but also for consumers, in order to provide full information about the nutritional value of the product and the daily dose a bioelement.

The aim of the study was to determine and compare the content of some micronutrients and macronutrients in fruits of some various species or cultivar varieties and hybrids of raspberries, i.e., from the species *Rubus occidentalis* in cultivar varieties ‘Bristol’, ‘MacBlack’, ‘Jewel’, ‘Niwot’, ‘Heban’, and hybrids: R1613411, R1613409, R1613412, and R1314701, from the *Rubus idaeus* in cultivar varieties ‘Delniwa’, ‘Husaria’, ‘Poranna Rosa’, ‘Promyk’, ‘Jantar’ and one hybrid, R1616002, and two other species, namely *Rubus chamaemorus* and *Rubus chingii.* In addition, chemometric tools were used to assess the diversity of the determined profile of the bioelements in raspberry fruits depending on the geographical environment and the species or its cultivar.

## 2. Materials and Methods

### 2.1. Plant Material

The plant material comprised fresh fruits from four cultivar varieties of *Rubus occidentalis* L. (*Rosaceae*), namely, ‘MacBlack’, ‘Jewel’, ‘Niwot’, ‘Heban’, and four its hybrids R1613411, R1613409, R1613412, R1314701; six cultivars of *Rubus idaeus* L. (*Rosaceae*), with yellow fruits—the varieties of ‘Poranna Rosa’, ‘Promyk’, ‘Jantar’ and red fruits—the varieties of ‘Delniwa’ and ‘Husaria’ and its one hybrid, R1616002 were collected in 2020 from plants growing at the Niwa Berry Breeding Company (Brzezna, Poland). Fruits of the ‘Bristol’ variety were obtained from two producers: “Czarna malina” Barbara Rusiecka-Górniak (Nałęczów, Poland) and BiGrim Grzegorz Maryniowski (Łaziska, Poland) in 2020. In addition, fresh fruits of *Rubus chamaemorus* L. (*Rosaceae*) were obtained from a commercial source in Finland and from plants cultivated by the producer Lubelskie Zioła (Sosnówka, Poland). The tested fruits were frozen at −20 °C, freeze-dried, and powdered. *Rubus chingii* L. (*Rosaceae*) dried fruits were purchased from commercial sources from China (Table 1). The plant material was stored without access to moisture at room temperature.

### 2.2. Preparation of Samples

A sample of 0.5 g (±0.0001 g) of powdered raspberry fruits were weighed in triplicate (*n* = 3) and then transferred to quartz crucibles and incinerated in an electric furnace (540 °C). The obtained fruit ash of various raspberry species and cultivars was mineralised with a mixture of concentrated acids. A sample of 1.50 mL 36% HCl and two to three drops of 63% HNO were added to ashes. After evaporating to dryness on a water bath, 1.50 mL 36% HCl was added and evaporated for 1 min under a watch glass. The solution was transferred to 25-mL flasks with ultrapure water from a Mili-Q system (18.2 MΩ·cm, Millipore Simplicity System, Billerica, MA, USA) [55].

### 2.3. Method

Elemental analyses of Ca, Mg, Na, K, Mn, Cu, Zn, Fe, Cr, Pb, and Cd were carried out using the atomic absorption spectrometry method with an air-acetylene flame (FAAS) and a background deuterium correction [56]. They were performed using Thermo Scientific’s iCE3000 (Waltham, MA, USA) instrument. In the case of Na and K, caesium chloride (0.2% *w*/*v*, Merck, Rahway, NJ, USA) was used as an ionisation buffer, which shifts the equilibrium of the reaction to produce free atoms of the element. Lanthanum (III) oxide (0.4% *w*/*v*, Merck), which acts as a corrective buffer and allows binding of the analysed element to a matrix, was used for the determination of Ca and Mg [55].

### 2.4. Validation of Method

The limit of detection (LOD) and limit of quantification (LOQ) for the elements analysed were calculated based on the standard solutions’ measurements and the calibration curves using the following formulas [57]:

$$LOD=\frac{3.3\times s}{b}$$

LOQ=3×LOD,

*s*—Standard deviation value; *b*—Slope of the calibration curve.

The accuracy and precision of the method were determined using a certified reference material, i.e., Oriental Basma Tobacco Leaves INCT-OBTL-5. The material was prepared according to the procedure applied for the determination of the analysed samples. Validation parameters are presented in Table 2.

### 2.5. Statistical Analysis

The Shapiro–Wilk test was used to assess the normal distribution of the data obtained [58]. Since the data were not normally distributed, non-parametric tests such as the Kruskal–Wallis test and Spearman’s rank correlations analysis were used. The data were subjected to factor analysis (FA) and cluster analysis (CA) using Statistica 13.3 software. The standardised data were arranged into columns–elements and rows–raspberry samples. The obtained database was used for statistical analyses in terms of species diversity and geographical environment of all the analysed samples, but also in view of species diversity and their origin only from Polish regions. Ward’s method and Euclidean distance, as a measure of distance between objects, were used in cluster analysis (CA).

## 3. Results and Discussion

### 3.1. Contents of Macro- and Micro-Elements in the Analysed Raspberry Fruits

The content of macroelements K, Ca, Na and Mg as well as microelements Cr, Zn, Fe, Mn, Cu, and heavy metals Cd and Pb were determined in the tested fruits of four raspberry species, namely *R. occidentalis*, *R. idaeus*, *R. chamaemorus*, and *R. chingii*, and their varieties (‘Bristol’,’Heban’, ‘Jewel’, ‘Niwot’, and ‘MacBlack’ from *R. occidentalis*, ‘Delniwa’, ‘Promyk’, ‘Poranna Rosa’, ‘Husaria’, and ‘Jantar’ from *R. idaeus*), and hybrids (*R. idaeus* R1616002, *R. occidentalis*/*R. idaeus* R1613411, *R. idaeus*/*R. occidentalis* R1613409, *R. occidentalis*/*R. idaeus* R1613412, and *R. idaeus*/*R. occidentalis* R1314701).

The obtained results confirm the high content of potassium (K) in raspberries, especially in all *Rubus idaeus* yellow fruit cultivars (1266.25–1374.74 mg/100 g) (Table 3) [48,49,50,51,52,53,54].

For comparison, in Russian varieties of yellow raspberries, 3–4 times lower concentrations of potassium were found [48].

On the other hand, among the analysed raspberries, the highest content of K was found in the red fruits of the R1616002 cultivar variety of *R. idaeus* (1460.19 mg/100 g) (Table 3). Among the analysed fruits of black raspberry (*Rubus occidentalis*), which were characterised by slightly lower potassium content (933.14–1040.46 mg/100 mg), the exception was the ‘Bristol’ variety. Its fruits contained either high (1140.27 mg/100 g) or relatively low (648.73 mg/100 g) potassium, depending on the growing region (Table 3). These differences in K levels in ‘Bristol’ cultivar fruits can be explained by different growing locations, including soil type and different growing conditions resulting from the use of different fertilisation systems (e.g., using K_2_O as a fertiliser ingredient). Literature data confirm the high level of K, in the previously tested black raspberry fruits of two varieties, ‘Bristol’ and ‘Jewel’ [50,51]. Similarly, in cloudberries (*Rubus chamaemorus*) the level of K was also relatively high (1023.51 and 1046.54 mg/100 g), regardless of the country of origin and cultivation (Table 3). It should be emphasised, that for the two analysed cloudberries, originating from different geographical regions (Finland and Poland), the profiles of bioelements, including K, were very similar. This can be attributed to the fact, that the conditions for cloudberry cultivation in Poland in an agroforestry system are used, which reproduce the conditions of its natural habitat in Finland (soil pH 3.5–2.5 and appropriate soil moisture characteristic for peat bogs) [59,60,61]. The analysed *Rubus chingii* fruits were also characterised by a high level of potassium (1273.07 mg/100 g) (Table 3). It is now known that there is a relationship between some elements suggesting their antagonism (K/Na) or synergism (Cr/Zn) [62], which is also confirmed by the results of our research (Table 3). It is obvious that if the concentrations of K are high in the plant material tested, then the concentrations of Na are low, for example, in shoots of *R. chamaemorus* and *Vaccinium uliginosum* [62]. All analysed raspberry fruits were characterised by high levels of K content compared to very low levels of Na content (Table 3). High Zn content is correlated with higher Cr concentration [62]. The increasing level of Zn, starting from cloudberry fruits through red and black raspberry fruits, was correlated with the increasing Cr content, the highest in the fruits of *R. occidentalis* cultivar ‘Bristol’ (Table 3). The levels of Fe and Zn content increased in a similar order, reaching the highest concentration among the fruits of *R. idaeus* and *R. occidentalis* cultivars, and hybrids, in the fruits of the ‘Bristol’ cultivar (Fe/Zn (synergistic relationship) (Table 3). The synergistic effect of Fe and Zn accumulation was also found in raspberry leaves [63]. This positive relationship contrasts with some previous literature data showing Fe/Zn antagonism [62,64].

Another macroelement found in relatively high amounts, ranging from 95.41 to 224.08 mg/100 g, was magnesium (Mg). Therefore, the consumption of 100 g of lyophilised raspberries can cover 26.9–70% of its daily requirement (RDA) (Table 4).

The highest Mg content was found in both cloudberries (184.11 and 215.77 mg/100 g) and in most fruits of black raspberry varieties: ‘Heban’ (224.08 mg/100 g), ‘Niwot’ (174.68 mg/100 g) and its hybrid R1613411 (190.78 mg/100 g). Comparing the determined content of Mg in the analysed black raspberry fruits with the literature data revealing the high content of Mg in fruits of *R. occidentalis* ‘Jewel’ and ‘Bristol’ cultivars, it should be stated that generally, black raspberry fruits are characterised by a high content of this element [50,51]. Mg is of great importance in glucose metabolism. Diabetes, on the other hand, leads to increased loss of Mg in the urine, and the resulting Mg deficiency can impair insulin secretion and action, thereby worsening diabetes control [65].

All analysed raspberry fruits were characterised by low concentrations of sodium (Na) (0.32–18.72 mg/100 g) and calcium (Ca) (1.31–30.97 mg/100 g) (Table 3). These contents only cover 0.5–3% of the daily requirement for Ca (Table 4). The low levels of Na in the investigated plant materials are consistent with the literature data [1,48]. On the other hand, the Ca content previously described in the literature for two cultivars of *R. occidentalis* ‘Jewel’ and ‘Bristol’ was 10 times higher than that determined in the analysed raspberries [50,51]. Calcium has been shown to protect against colon cancer in humans and chemically induced colon cancer in animals through a variety of mechanisms, including complexation of cancer-promoting bile acids and inhibition of cell proliferation [40]. Excessive amounts of sodium can cause high blood pressure, so low-sodium products are best for patients, especially those with hypertension [66].

The results of microelement determinations showed large differences in the manganese (Mn) content in the analysed raspberries, ranging from 0.13 to 8.45 mg/100 g (Table 3). Both fruits of the ‘Bristol’ variety were characterised by a high content of Mn–6.91 and 8.45 mg/100 g. The lowest content of Mn was found in the yellow fruits of the variety *R. idaeus* ‘Jantar’ (0.13 mg/100 g). However, in the remaining yellow raspberries, a higher level of Mn was found (varieties *R. idaeus* ‘Promyk’ 0.40 mg/100 g and ‘Poranna Rosa’ 0.66 mg/100 g, and both *Rubus chamaemorus* 0.59 and 0.63 mg/100 g) (Table 3). Earlier studies revealed levels of Mn in raspberry fruits in the range of 2.3–3.68 mg/100 g [1,50,51,52,54]. In studies comparing the content of Mn in various fruits, it was shown that the content of this bioelement in red raspberry juice was 10 times higher than in blackcurrant and redcurrant juices, covering 50% of the daily requirement [67]. Mn is an essential bioelement and co-factor for many enzymes, such as pyruvate kinase, superoxide dismutase, and xanthine oxidase, involved in the metabolism of carbohydrates, proteins, lipids, and many defense mechanisms [68]. Mn deficiency, as well as its excess, can be harmful to health. Dietary references indicate that the daily intake of this bioelement should be 1.8 mg/person for women and 2.3 mg/person for men, although these values change with age [69].

The presence of chromium (Cr) was found in all raspberry fruits, regardless of the species. Cr was present in the highest concentrations in the fruits of black raspberry ‘Niwot’ (0.089 mg/100 g) and both ‘Bristol’ (0.077 mg/100 g) (Table 3). The presence of Cr in black raspberry fruits was demonstrated for the first time. Moreover, *Rubus chingii* fruits were also characterised by a relatively high Cr content (0.062 mg/100 g). Cloudberry fruits contained the least Cr (0.011 and 0.012 mg/100 g), similar to Zn and Cu, present in these fruits in the lowest concentrations, compared to other fruits. Studies on Cr deficiency indicate that chromium is an essential element involved in the action of insulin [67]. There is increasing evidence that Cr supplementation, especially in higher doses and in the form of free chromium, may improve insulin and glucose sensitivity in type 1 and 2 diabetes [70,71]. The ‘Bristol’ variety of black raspberry seems to be important in the prevention and treatment of metabolic diseases due to the high content of chromium, zinc and manganese, the deficiency of which is associated, among others, with the development of diabetes.

The highest content of iron (Fe) was found in the fruits of the *Rubus chingii* (10.22 mg/100 g) and the ‘Bristol’ cultivar variety (5.15–6.08 mg/100 g). The fruits of other *R. occidentalis* varieties contain 2–3.5 times less Fe (‘MacBlack’ 1.60 mg/100 g; ‘Heban’ 1.96 mg/100 g; ‘Niwot’ 2.24 mg/100 g; ‘Jewel’ 2.43 mg/100 g). Both the red and yellow *Rubus idaeus* fruits contained Fe at a similar level to that of black raspberries, with the exception of the variety ‘Poranna Rosa’ with yellow fruits (4.27 mg/100 g) (Table 3). Literature data [49] indicate a similar Fe content in both red raspberries (3.1–3.5 mg/100 g) and black raspberries (4.82 mg/100 g), while the Fe content in the analysed fruits of the ‘Bristol’ variety is marked twice as high [50,51]. Similarly to copper, chromium, and zinc, cloudberries contain the least iron compared to other raspberry species and varieties (0.25 and 0.28 mg/100 g). Iron, a redox-active metal, plays an important role in the antioxidant defense system [71]. Raspberries belong to the second group of fruits with the highest vitamin C content, which increases the absorption of non-haem iron [72].

The fruit of *Rubus chingii* (6.84 mg/100 g) was characterised by a high concentration of zinc (Zn), in contrast to both cloudberries with the lowest content of this microelement (0.33 and 0.35 mg/100 g) (Table 3). The performed analysis showed the content of Zn at a similar level in the fruits of all tested varieties of *Rubus idaeus* and *R. occidentalis* (1.58–3.07 mg/100 g), which was consistent with the literature data (1.9–2.69 mg/100 g) [48,49,50]. Zn is an element with an insulin-mimetic effect and plays an important role in regulating blood glucose levels. Additionally, it protects cells from oxidative damage [73].

The highest Cu content was found in the fruits of *R. chingii* (17.42 mg/100 g) and the lowest in cloudberries (0.05 and 0.07 mg/100 g) (Table 3). In addition, a high content of copper (Cu) was revealed in the fruits of red and black raspberries, covering from 80% (*R. idaeus* ‘Husaria’ 0.72 mg/100 g, ‘Delniwa’ 0.77 mg/100 g and R1616002 0.77 mg/100 g) to 132% (*R. occidentalis* ‘Bristol’ 1.19 and 1.28 mg/100 g) of the daily requirement for this bioelement. The determined contents were about twice as high as those reported previously in the fruits of the ‘Bristol’ and ‘Jewel’ varieties (0.56–0.86 mg/100 g) [48,49,50]. Cu plays a key role in the functioning of the nervous system, and it can act preventively in neurodegenerative diseases such as Alzheimer’s disease [74].

Raspberry fruits, due to the rich complex of polyphenols [10,24], are classified as superfoods, and the content of bioelements determined in them guarantees coverage of the daily requirement for macro- and microelements (RDA depending on the element: 5.6–204% for *R. idaeus*, 8.8–469.4% for *R. occidentalis* and 1.4–67.2% for *R. chamaemorus*) (Table 4), finally confirming this opinion.

### 3.2. Correlations

The non-parametric Spearman’s rank test was used at three significance levels, i.e., *p* < 0.05, *p* < 0.01, and *p* < 0.001. Spearman’s rank correlations measure the strength and direction of the relationship between two ranked variables. There were found negative and positive correlations between the analysed elements in all three datasets. The positive correlations (*p* < 0.001) were found in the database of all the analysed samples between the following pairs of elements: Ca-Zn, Ca-Cu, Ca-Mn, Ca-Cr, Fe-Zn, Fe-Cu, Fe-Mn, Zn-Cu, Zn-Mn, and Cu-Mn. Additionally, Spearman’s rank analysis was also performed only for samples from Poland. The existence of positive correlations (*p* < 0.001) was confirmed in the case of Ca and Cu, Ca-Mn, Fe-Zn, Zn-Cu, Zn-Mn, and Cu-Mn. Similar positive correlations between Fe and Zn were observed in the study by Dresler [64]. They found a positive correlation between Fe and Zn in raspberry leaves with a concomitant increase in K in the soil. In addition, the authors also suggest that elevated Mn content due to soil conditions can also affect the concentration of other elements that are accumulated by the plant [64].

### 3.3. Kruskal–Wallis Test

The Kruskal–Wallis test showed statistically significant differences in the analysed database. The dataset was divided into four categories, i.e., first, all samples in view of their species; second, all samples from different geographical provenances; third, in view of species of Polish origin; and fourth, raspberry samples only of Polish cultivation region. Relationships between the raspberry species (first category) and the concentration of elements were as follows: Mg (H = 8.092; *p* = 0.151), K (H = 20.523; *p* = 0.001), Na (H = 34.845; *p* = 0.000), Ca (H = 46.129; *p* = 0.000), Fe (H = 23.119; *p* = 0.000), Zn (H = 26.153; *p* = 0.000), Cu (H = 30.969; *p* = 0.000), Mn (H = 42.802; *p* = 0.000), Cr (H = 18.612; *p* = 0.002), and Pb (H = 11.088; *p* = 0.050). The Kruskal–Wallis test revealed relationships between the geographical environment of all raspberry (second category) samples and the concentration of elements, which were as follows: Mg (H = 10.350; *p* = 0.066), K (H = 12.706; *p* = 0.026), Na (H = 21.465; *p* = 0.001), Ca (H = 23.990; *p* = 0.000), Fe (H = 33.490; *p* = 0.000), Zn (H = 31.680; *p* = 0.000), Cu (H = 33.522; *p* = 0.000), Mn (H = 23.486; *p* = 0.000), Cr (H = 19.443; *p* = 0.002), and Pb (H = 13.657; *p* = 0.018).

The Kruskal–Wallis test was also performed for raspberry samples originating only from Poland. The interdependences between the concentrations of elements and species of raspberry (third category) samples of Polish origin were as follows: Mg (H = 4.772; *p* = 0.311), K (H = 16.070; *p* = 0.029), Na (H = 27.146; *p* = 0.000), Ca (H = 37.773; *p* = 0.000), Fe (H = 9.090; *p* = 0.060), Zn (H = 12.871; *p* = 0.012), Cu (H = 18.876; *p* = 0.001), Mn (H = 36.782; *p* = 0.000), Cr (H = 9.683; *p* = 0.046) and Pb (H = 11.704; *p* = 0.020). Relationships between the cultivation region of raspberry samples from Poland (fourth category) and the elemental content were as follows: Mg (H = 7.274; *p* = 0.064), K (H = 8.871; *p* = 0.031), Na (H = 11.671; *p* = 0.009), Ca (H = 10.114; *p* = 0.018), Fe (H = 22.010; *p* = 0.000), Zn (H = 19.754; *p* = 0.000), Cu (H = 21.998; *p* = 0.000), Mn (H = 16.161; *p* = 0.001), Cr (H = 9.978; *p* = 0.019), and Pb (H = 6.643; *p* = 0.084). The Kruskal–Wallis test performed allowed us to indicate the presence of statistically significant differences between one group and the others. These differences were indicated in terms of raspberry species and its geographical environment. The next step was to perform a post-hoc test, the Dunn test, to indicate which specific averages were statistically significant compared to the other groups of data.

### 3.4. Post Hoc Dunn’s Test

The post hoc test used, i.e., Dunn’s test, was conducted to determine which averages were more significant than the others. The results of the Dunn test for all the samples analysed by species are shown in Table 5.

Table 6 presents the results of the Dunn’s test for all the raspberry samples, in view of their geographical environment. This test was performed at three levels of significance: *p* < 0.05, *p* < 0.01, and *p* < 0.001.

Significant relationships (*p* < 0.001) were found for Na, Ca, Mn, Cu, Zn, and Fe and *Rubus occidentalis*, *Rubus chingii*, and *Rubus chamaemorus* species (Table 5). The place of cultivation of raspberry samples such as Nałęczów (Lublin Voivodship/Poland), China, Łaziska (Lublin Voivodship/Poland), Finland, and Sosnówka (Lublin Voivodship/Poland) (*p* < 0.01) were associated with Na, Ca, Fe, Zn, Cu, Cr, and Pb concentrations.

Raspberry samples cultivated only in Polish regions were also analysed by Dunn’s test (*p* < 0.05, *p* < 0.01, and *p* < 0.001) in view of species and region of cultivation. Significant relationships for Na, Ca, and Mn in *R. occidentalis*, *R. idaeus*, and *R. idaeus*/*R. occidentalis* were determined (Table 7). Statistically significant relationships were found between concentrations of Zn, Cu, and Fe and raspberry samples from Nałęczów (Lublin Voivodship/Poland), Łaziska (Lublin Voivodship/Poland), and Sosnówka (Lublin Voivodship/Poland) (Table 8).

### 3.5. Factor Analysis

**Figure 1:**
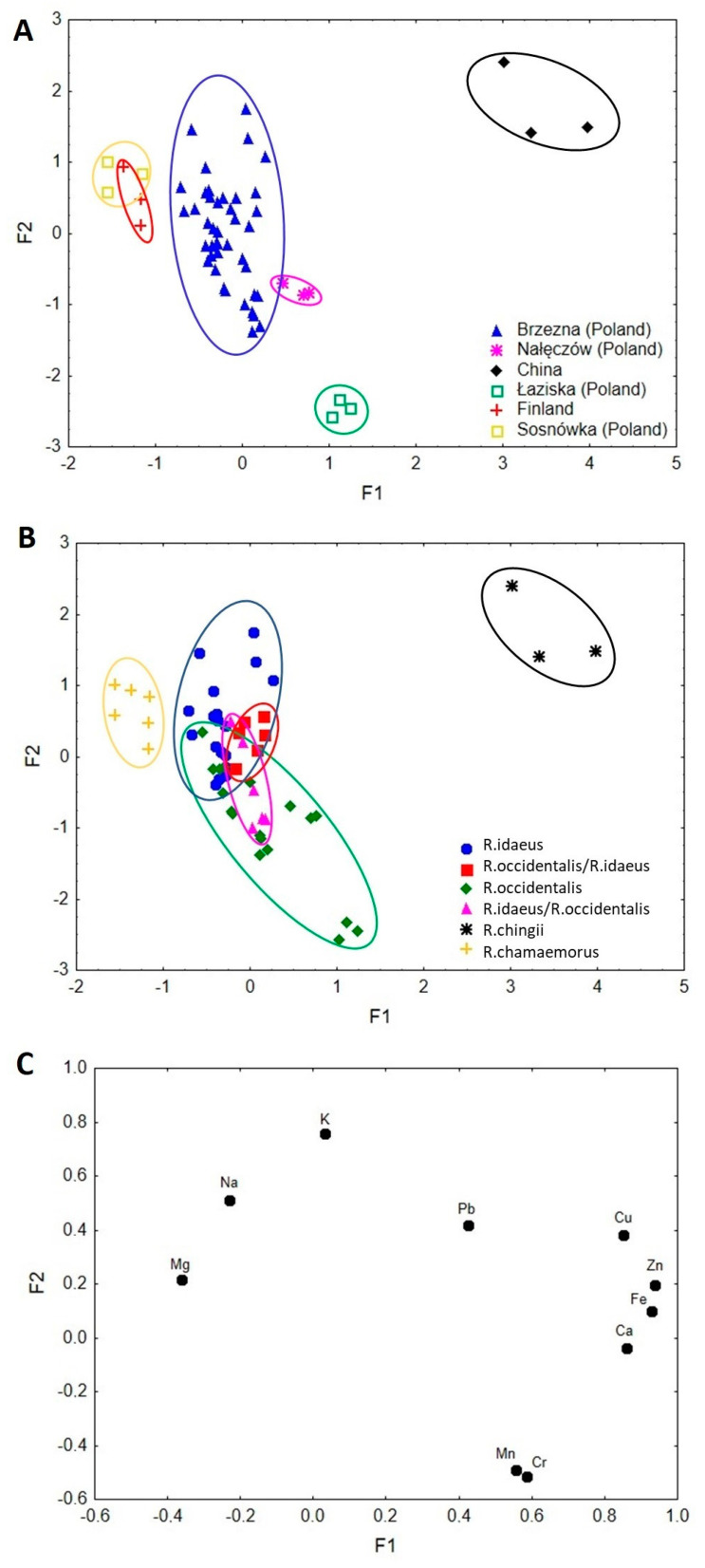
(**A**) Scatterplot of object samples of two factors of all raspberry geographical environments. (**B**) Scatterplot of object samples of two factors of all raspberry species. (**C**) Scatterplot of loadings for elements in all the analysed samples.

First, a factor analysis was performed for all the analysed samples of all raspberry species and of various geographical environments. The results are presented in Figure 1A–C.

All the elements were taken into consideration. The value of the first factor (F1) of the explained variance amounted to 42.2%, while of the second factor (F2) to 17.6%. Both factors cumulatively explained 59.8% of the total variance, whereas the eigenvalues for F1 and F2 were 4.22 and 1.76, respectively.

As can be seen in Figure 1A, F1 distinguishes samples based on their geographical environment. The lowest values of F1 corresponded to raspberry samples from Sosnówka (Lublin Voivodship/Poland) and Finland, described by Na and Mg, respectively. Potassium and Pb were descriptors of Brzezna (Lasser Poland) samples. Higher values of F1 distinguished samples from China, whose descriptors were Cu, Zn, Fe, and Ca. Raspberry samples from Łaziska (Lublin Voivodship/Poland) and Nałęczów (Lublin Voivodship/Poland) were characterised by Mn and Cr (Figure 1C). Factor 2 was responsible for the distribution of samples from Łaziska (Lublin Voivodship/Poland) and Nałęczów (Lublin Voivodship/Poland) described by Mn and Cr. Samples from China corresponded to Ca, Fe, Zn, and Cu, Sosnówka (Lublin Voivodship/Poland) to Na, Finland to Mg, and Brzezna (Lasser Poland) to K and Pb.

Figure 1B,C present the same scatterplot but classify samples in view of raspberry species. Within the low values of F1, there are distinguished *R. chamaemorus* (Na and Mg), *R. occidentalis* (Cr), *R. idaeus* (K), *R. occidentalis*/*R. idaeus* (Pb), and *R. idaeus*/*R. occidentalis* (Mn) from *R. chingii* (Cu, Zn, Fe, and Ca) (Figure 1C). Lower values of F2 were responsible for the distribution of *R. occidentalis* and *R. idaeus*/*R. occidentalis*, which corresponded to Mn and Cr. Lead was the descriptor of *R. occidentalis*/*R. idaeus*. Higher values of F2 described *R. chingii* (Cu, Zn, Fe, and Ca), *R. chamaemorus* (Na and Mg), *R. idaeus* (K), and *R. occidentalis*/*R. idaeus* (Pb). This factor analysis allowed diversification of the analysed raspberry samples in view of the geographical environment and the species.

**Figure 2:**
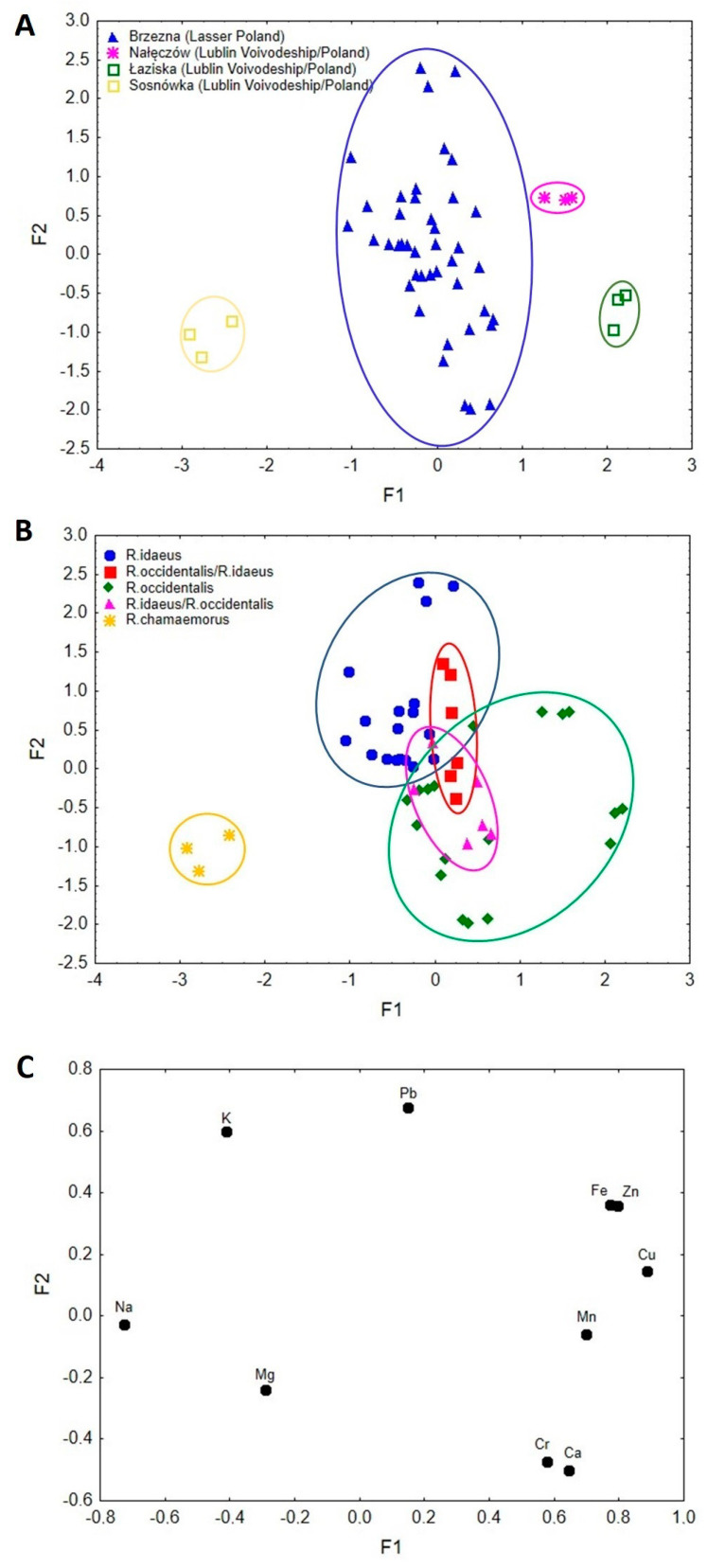
(**A**) Scatterplot of object samples of two factors of the Polish raspberry cultivation regions. (**B**) Scatter plot of object samples of two factors of the Polish raspberry species. (**C**) Scatterplot of loadings for elements in all the analysed samples.

Factor analysis of raspberry samples from Poland allowed the diversification of samples in view of cultivation region and species (Figure 2A–C).

It was found that 56.8% of the total variance was explained by F1 (40.5%) and F2 (16.2%). The eigenvalues were 4.05 and 1.62 for F1 and F2, respectively. Lower values of F1 described samples from Sosnówka (Lublin Voivodship/Poland), which corresponded to Na. Samples from Brzezna (Lasser Poland) were characterised by Ca, K, Mg, Cr, and Pb. Higher values of F1 distinguished samples from Nałęczów (Lublin Voivodship/Poland) and Łaziska (Lublin Voivodship/Poland), which corresponded to Fe, Zn, Cu, and Mn (Figure 2C). Sosnówka (Lublin Voivodship/Poland), Łaziska (Lublin Voivodship/Poland), and partially Brzezna (Lesser Poland) were characterised by lower values of F2 described by Na, Mn, Cu, and Fe. Higher values of F2 corresponded to samples from Nałęczów (Lublin Voivodship/Poland)–Zn and Fe and partially Brzezna (Lesser Poland)–K and Pb. The same scatterplots were presented in Figure 2B,C in view of raspberry species. Factor 1 separated *R. chamaemorus* (Na) from *R. idaeus*, *R. occidentalis*, *R. occidentalis*/*R. idaeus*, and *R. idaeus*/*R. occidentalis* (Ca, K, Mg, Cr, Pb, Zn, Mn, Cu, and Fe). Lower values of F2 described *R. occidentalis* samples (Cr and Ca) and *R. chamaemorus* (Na and Mg), whereas higher values of F2 corresponded to *R. idaeus* samples (K and Pb). *R. occidentalis*/*R. idaeus* samples were described by Fe and Zn, while *R. idaeus*/*R. occidentalis* by Mn.

Factor analysis showed that the hybrid R1613409 with dark purple fruits, described by the breeder as a hybrid with dominant attributes of *R. idaeus (R. idaeus*/*R. occidentalis*, Table 1) has a bioelement profile characteristic of black raspberry (Table 3, Figure 1B and Figure 2B) and can be included in the cultivated varieties of black raspberry, also due to the complex of anthocyanins, characteristic of black raspberry fruit (unpublished data). On the other hand, the belonging of hybrid R1613412 with purple fruits to *R. occidentalis* cultivars (*R. occidentalis*/*R. idaeus*, Table 1) was not confirmed by the profile of bioelements (Table 3, Figure 1B and Figure 2B), while the quality profile of anthocyanins was characteristic of black raspberry (unpublished data). On this basis, it can be suggested that factor analysis can be a useful tool in determining the affiliation of a given hybrid or cultivar to one of the parent species.

### 3.6. Cluster Analysis

Cluster analysis was performed using Ward’s method and the Euclidean distance.

**Figure 3:**
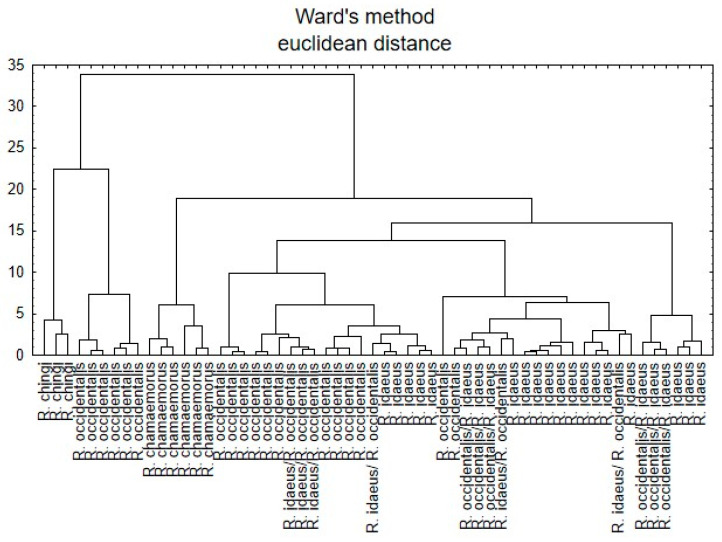
Hierarchical dendrogram for all raspberry samples.

CA analysis was also performed on all raspberry sample species. Figure 3 presents the dendrogram, which shows differentiation into six main clusters.

Both FA and CA analysis showed a high similarity between samples of the same species (*R. chamaemorus*), but from different geographical environments (Finland and Sosnówka, Poland), with and a clear separation of the species (*R. chingii*) from China.

## 4. Conclusions

A similar qualitative composition of bioelements in raspberry fruits from different species, varieties, and hybrids was revealed (Table 3). The differences relate to the levels of the content of individual bioelements in the tested fruits, which is associated with differences in the coverage of the daily requirement (RDA, after consuming 100 g of lyophilised raspberries and depending on the element), respectively for given species in the range of 5.6–204% for *R. idaeus*, 8.8–469.4% for *R. occidentalis* and 1.4–67.2% for *R. chamaemorus* (Table 4). The analysed fruits of various raspberry species, their cultivars, and hybrids turned out to be a rich source of K and Mg and a low source of Ca and Na. Among the tested microelements, large differences were found in the content of Mn up to 16 times more in some raspberry fruits (Table 3). The fruits of *R. occidentalis* and *R. idaeus*, in contrast to fruits of *R. chamaemorus*, contained higher concentrations of Zn, Cu, and Cr (Table 3). The presence of Cr in black raspberry fruits was demonstrated for the first time. Among the analysed fruits of various species and cultivar/varieties of raspberries, black raspberry fruits, especially the ‘Bristol’ variety, are characterised by the highest contents of macroelements such as K, Mg, and microelements such as Cr, Mn, Zn, Cu and Fe (Table 4). In the tested raspberry fruits, the antagonistic effect of K/Na, as well as the synergistic effect of Cr/Zn and Fe/Zn, described earlier in the literature [62,63], were confirmed.

Both FA and CA analyses showed the species, cultivar, and hybrid diversity of raspberry samples and the diversity of samples by geographical environment (Figure 1, Figure 2 and Figure 3). On the other hand, FA and CA showed a high similarity between fruits of *R. chamaemorus*, originating from different geographical environments (Finland and Poland). With regard to the obtained FA results, it is suggested that factor analysis may be a useful tool in determining the affiliation of a given hybrid or variety to one of the parent species.

Taking into account the physiological and biochemical importance of individual bioelements [32,33,34,35], it can be concluded that the consumed raspberry fruits, covering the daily requirement for macro- and microelements (Table 4), can be an important component of the daily diet and prevent such civilisation diseases like diabetes or metabolic syndrome, and even support their treatment [44,45] (further research is needed).

## Figures and Tables

**Table 1:** Characteristics of the analysed fruits from different raspberry species and cultivars or hybrids (parent plants, colour of fruit, origin).

Cultivar or Hybrids/Colour of Fruit	Origin of the Cultivar	Pedigree * Op–Open-Pollination
*R. occidentalis* ‘Jewel’/black	Niwa Berry Breeding Ltd. (Brzezna, Poland)	‘Dundee’ x (‘Bristol’ x ‘Dundee’)
*R. occidentalis* ‘Niwot’/black	Niwa Berry Breeding Ltd. (Brzezna, Poland)	A complex cross between two breeding clones from a natural environment of origin in the USA
*R. occidentalis* ‘MacBlack’/black	Niwa Berry Breeding Ltd. (Brzezna, Poland)	-
*R. occidentalis* ‘Heban’ (R139501)/black	Niwa Berry Breeding Ltd. (Brzezna, Poland)	(purple raspberry x ‘Polka’) x op
*R. occidentalis* ‘Bristol’ A/black	“Czarna malina” Barbara Rusiecka-Górniak (Nałęczów, Poland)	-
*R. occidentalis* ‘Bristol’ B/black	“BiGrim” Grzegorz Maryniowski (Łaziska, Poland)	-
*R. idaeus* ‘Husaria’/red	Niwa Berry Breeding Ltd. (Brzezna, Poland)	R120701 x ‘Sokolica’
*R. idaeus* ‘Delniwa’/red	Niwa Berry Breeding Ltd. (Brzezna, Poland)	‘Polka’ x R1211101
*R. idaeus* ‘Poranna rosa’/yellow	Niwa Berry Breeding Ltd. (Brzezna, Poland)	89112(83291 x ‘ORUS’ 10 98-1)
*R. idaeus* ‘Jantar’/yellow	Niwa Berry Breeding Ltd. (Brzezna, Poland)	R126107 (‘Heritage’ x ‘Polesie’)
*R. idaeus* ‘Promyk’/yellow	Niwa Berry Breeding Ltd. (Brzezna, Poland)	‘Poemat’ x R127302 (‘Pingvin’ x op)
*R. occidentalis*/*R. idaeus* R1613411/Purple	Niwa Berry Breeding Ltd. (Brzezna, Poland).	‘Jewel’ x R121304 (‘Litacz’ (‘Bristol’ x op) x purple raspberry x op)
*R. idaeus*/*R. occidentalis* R1613409/dark purple	Niwa Berry Breeding Ltd. (Brzezna, Poland)	‘Jewel’ x R121304 (purple raspberry x op)
*R. occidentalis*/*R. idaeus* R1613412/purple	Niwa Berry Breeding Ltd. (Brzezna, Poland)	‘Jewel’ x R121304 (‘Litacz’ x purple raspberry) x op
*R. idaeus*/ *R. occidentalis* R1314701/purple	Niwa Berry Breeding Ltd. (Brzezna, Poland)	‘Litacz’ x ‘Sokolica’
*R. idaeus* R1616002/red	Niwa Berry Breeding Ltd. (Brzezna, Poland)	R1634401 x ‘Polana’
*R. chamaemorus*	Commercial source, Finland	-
*R. chamaemorus*	Lubelskie Zioła, Poland (Sosnówka, Poland).	-
*R. chingii*	Commercial source, China	-

* data provided by the breeder Niwa Berry Breeding Ltd.

**Table 2:** Analytical method validation parameters for each element: limit of detection (LOD), limit of quantification (LOQ), recovery, and relative standard deviation (RSD) using a certified reference material (Oriental Basma Tobacco Leaves INCT-OBTL-5).

Element	LOD (µg/mL)	LOQ (µg/mL)	Recovery (%)	RSD (%)
Ca	0.0001	0.0003	92	4.15
Cu	0.0002	0.0006	97	1.42
Cd	0.0002	0.0006	101	3.42
Cr	0.0002	0.0006	95	0.02
Mg	0.0005	0.0015	99	0.59
Mn	0.0001	0.0003	108	1.57
Zn	0.0001	0.0003	104	3.31
K	0.0005	0.0015	102	2.06
Na	0.0002	0.0006	98	10.1
Pb	0.0002	0.0006	92	4.50
Fe	0.0002	0.0006	101	0.62

**Table 3:** Determined contents of macro- and microelements in the analysed raspberry fruits, mg/100 g (*n* = 3) (mean ± SD).

Species/Variety/Hybrid	Mg	Ca	Na	K	Fe	Zn	Cr	Cu	Mn	Pb	Cd
*Rubus idaeus*/ ‘Poranna Rosa’	126.22 ± 5.44	9.33 ± 0.84	6.11 ± 0.84	1374.74 ± 14.3	4.27 ± 0.33	2.68 ± 0.06	0.025 ± 0.004	0.85 ± 0.02	0.66 ± 0.01	0.0299 ± 0.0015	ND
‘Promyk’	138.34 ± 3.12	3.96 ± 0.06	7.96 ± 0.4	1373.69 ± 3.77	2.84 ± 0.16	1.82 ± 0.04	0.051 ± 0.004	0.84 ± 0.02	0.4 ± 0.03	0.012 ± 0.0008	ND
‘Jantar’	168.64 ± 1.28	7.79 ± 0.35	3.3 ± 0.21	1266.25 ± 5.97	2.31 ± 0.21	2.29 ± 0.11	0.027 ± 0.007	1.01 ± 0.04	0.13 ± 0.02	0.0163 ± 0.001	ND
‘Delniwa’	113.63 ± 8.12	7.47 ± 0.9	3.99 ± 0.41	935.06 ± 8.42	2.76 ± 0.02	1.58 ± 0.13	0.032 ± 0.006	0.77 ± 0.18	0.16 ± 0.02	0.0176 ± 0.0004	ND
‘Husaria’	161.35 ± 8.75	5.03 ± 0.46	3.03 ± 0.27	1109.32 ± 15.14	2.88 ± 0.07	2.26 ± 0.12	0.042 ± 0.007	0.72 ± 0.21	0.17 ± 0.01	0.0124 ± 0.0006	0.009 ± 0.001
*Rubus occidentalis*/ ‘Jewel’	110.11 ± 6.13	19.02 ± 0.85	1.37 ± 0.23	1003.98 ± 7.92	2.43 ± 0.32	2.03 ± 0.22	0.019 ± 0.004	0.95 ± 0.09	0.84 ± 0.01	0.0123 ± 0.0009	0.086 ± 0.002
‘MacBlack’	134.05 ± 4.97	29.51 ± 2.27	0.32 ± 0.05	933.14 ± 1.78	1.6 ± 0.33	1.81 ± 0.19	0.046 ± 0.005	0.94 ± 0.11	0.73 ± 0.01	0.0124 ± 0.0004	ND
‘Niwot’	174.68 ± 10.6	30.97 ± 2.24	2.85 ± 0.22	969.65 ± 7.74	2.24 ± 0.06	1.86 ± 0.14	0.089 ± 0.009	0.86 ± 0.08	0.8 ± 0.02	0.0095 ± 0.001	ND
‘Heban’	224.08 ± 4.43	21.56 ± 1.05	1.54 ± 0.59	1040.46 ± 18.51	1.96 ± 0.05	2.12 ± 0.02	0.02 ± 0.007	0.94 ± 0.07	0.68 ± 0.02	0.0134 ± 0.0013	ND
‘Bristol’ A	148.36 ± 4.28	17.62 ± 0.38	1.27 ± 0.2	1140.27 ± 4.11	5.15 ± 0.06	3.07 ± 0.1	0.038 ± 0.012	1.28 ± 0.03	6.91 ± 0.01	0.0131 ± 0.0006	0.0119 ± 0.0015
‘Bristol’ B	95.41 ± 4.48	24.47 ± 0.61	2.83 ± 0.57	648.73 ± 0.85	6.08 ± 0.36	2.86 ± 0.02	0.077 ± 0.003	1.19 ± 0.05	8.45 ± 0.03	0.0135 ± 0.0008	0.0164 ± 0.0026
*R. occidentalis*/*R. idaeus*											
R1613411	190.78 ± 4.21	26.91 ± 1.66	2.58 ± 0.11	1221.16 ± 1.62	2.76 ± 0.1	2.62 ± 0.29	0.025 ± 0.001	0.98 ± 0.03	0.83 ± 0.06	0.0097 ± 0.0015	ND
R1613412	116.2 ± 5.45	19.31 ± 0.8	2.99 ± 0.04	986.07 ± 7.27	2.25 ± 0.37	2.38 ± 0.14	0.029 ± 0.004	0.9 ± 0.1	0.85 ± 0.01	0.0333 ± 0.0009	ND
*R. idaeus*/*R. occidentalis* R1613409	135.05 ± 7.31	27.47 ± 0.12	2.5 ± 0.12	819.93 ± 5.77	1.97 ± 0.24	2.76 ± 0.29	0.051 ± 0.005	0.97 ± 0.05	1.11 ± 0.04	0.0154 ± 0.0012	ND
R1314701	137.4 ± 6.61	21.39 ± 1.31	4.78 ± 0.91	1185.37 ± 2.94	2.39 ± 0.08	2.19 ± 0.12	0.04 ± 0.002	0.96 ± 0.08	0.81 ± 0.05	0.0137 ± 0.0008	0.0041 ± 0.0007
*R. idaeus*											
R1616002	163.53 ± 6.88	5.03 ± 0.15	4.09 ± 0.4	1460.19 ± 0.49	1.84 ± 0.03	1.71 ± 0.01	0.025 ± 0.01	0.77 ± 0.03	0.18 ± 0.01	0.0118 ± 0.0004	ND
*Rubus chingii*	122.52 ± 2.45	72.13 ± 6.84	7.29 ± 1.02	1273.07 ± 5.28	10.22 ± 0.11	6.84 ± 0.15	0.062 ± 0.019	17.42 ± 0.78	3.4 ± 0.08	0.0273 ± 0.0051	0.028 ± 0.009
*Rubus chamaemorus* Poland	184.11 ± 2.96	1.56 ± 0.17	18.72 ± 0.49	1046.54 ± 7.78	0.28 ± 0.06	0.33 ± 0.01	0.012 ± 0.001	0.05 ± 0.01	0.63 ± 0.03	0.0183 ± 0.0026	0.0233 ± 0.0011
*Rubus chamaemorus* Finland	215.77 ± 9.73	1.31 ± 0.02	8.63 ± 1.32	1023.51 ± 2.9	0.25 ± 0.1	0.35 ± 0.01	0.011 ± 0.001	0.07 ± 0.01	0.59 ± 0.11	0.0077 ± 0.0005	0.0105 ± 0.0013

ND—not detected, LOD for Cd = 0.0002.

**Table 4:** Comparison of (for a person weighing 70 kg through consumption of 100 g lyophilised raspberry fruits).

Element	Recommended Daily Allowance (RDA) (mg/Day/Person)		Average Content in 100 g *Rubus idaeus* Lyophilised Fruits	Percentage of RDA		Average Content in 100 g *Rubus occidentalis* Lyophilised Fruits	Percentage of RDA		Average Content in 100 g *Rubus chamaemorus* Lyophilised Fruits	Percentage of RDA	
	Male (31–50 Years)	Female (31–50 Years)		Male (31–50 Years)	Female (31–50 Years)		Male (31–50 Years)	Female (31–50 Years)		Male (31–50 Years)	Female (31–50 Years)
Mg	420	320	113–168	26.9–40	35–52.5	95–224	22.6–53.3	29.7–70	184–215	43.8–51.2	57.5–67.2
K	4700	4700	935–1460	19.9–31.1	19.9–31.1	648–1140	13.8–24.2	13.8–24.2	1023–1046	21.8–22.2	21.8–22.2
Fe	10	18	1.84–4.27	18.4–42.7	10.2–23.7	1.6–6.08	16–60.8	8.8–33.8	0.25–0.28	2.5–2.8	1.4–1.5
Zn	11	8	1.58–2.68	14.4–24.4	19.7–33.5	1.81–3.07	16.4–27.9	22.6–38.4	0.33–0.35	3–3.2	4.1–4.4
Cu	0.9	0.9	0.72–1.01	80–112.2	80–112.2	0.86–1.28	95.5–142.2	95.5–142.2	0.05–0.07	5.5–7.8	5.5–7.8
Mn	2.3	1.8	0.13–0.66	5.6–28.7	7.2–36.7	0.68–8.45	29.6–367.4	37.7–469.4	0.59–0.63	25.6–27.4	32.7–35
Cr ^a^	0.035	0.025	0.025–0.051	71.4–145.7	100–204	0.019–0.089	54.3–254.3	76–356	0.011–0.012	31.4–34.3	44–48

^a^ American recommendations.

**Table 5:** Results of the Dunn’s test conducted for the analysed data matrix concerning raspberry species (*p* < 0.05, *p* < 0.01, and *p* < 0.001).

	*R. idaeus*	*R. occidentalis*/ *R. idaeus*	*R. occidentalis*	*R. idaeus*/ *R. occidenatlis*	*R. chingii*	*R. chamaemorus*
*R. idaeus*	-	Ca ^a^, Mn ^a^	K ^b^, Na ^c^, Ca ^c^, Mn^c^	Ca ^a^, Mn ^b^	Ca ^b^, Cu ^a^, Mn ^b^	Fe ^b^
*R. occidentalis*/ *R. idaeus*	Ca ^a^, Mn ^b^	-				Na ^a^, Ca ^b^, Fe ^a^, Zn ^b^
*R. occidentalis*	K ^b^, Na ^c^, Ca ^c^, Mn ^c^		-		Na ^a^	Na ^c^, Ca ^c^, Fe ^a^, Zn ^b^, Cu ^c^, Cr ^a^
*R. idaeus*/ *R. occidenatlis*	Ca ^a^, Mn ^b^			-		Ca ^b^, Zn ^a^, Cu ^a^, Cr ^a^
*R. chingii*	Ca ^b^,Cu ^a^, Mn ^b^		Na ^a^		-	Ca ^c^, Fe ^c^, Zn ^c^, Cu ^c^, Cr ^a^
*R. chamaemorus*	Fe ^b^	Na ^a^, Ca ^b^, Fe ^a^, Zn ^b^	Na ^c^, Ca ^c^, Fe ^a^, Zn ^b^, Cu ^c^, Cr ^a^	Ca ^b^, Zn ^a^, Cu ^a^, Cr ^a^	Ca ^c^, Fe ^c^, Zn ^c^, Cu ^c^, Cr ^a^	-

^a^ *p* < 0.05. ^b^ *p* < 0.01. ^c^ *p* < 0.001.

**Table 6:** Results of the Dunn’s test conducted for the analysed data matrix concerning the raspberries’ geographical environment (*p* < 0.05 and *p* < 0.01).

	Brzezna/Lesser/ Poland	Nałęczów/Lublin Voivodship/Poland	China	Łaziska/Lublin Voivodship/Poland	Finland	Sosnówka/Lublin Voivodship/Poland
Brzezna/Lesser Poland	-			Mn^a^		Na^a^
Nałęczów/Lublin Voivodship/Poland		-			Na ^a^, Fe ^b^, Zn ^b^, Cu ^b^	Na ^b^, Fe ^b^, Zn ^b^, Cu ^b^
China			-	K ^a^	Ca ^b^, Fe ^b^, Zn ^b^, Cu ^b^, Cr ^a^	Ca ^b^, Fe ^b^, Zn ^b^, Cu ^b^, Pb ^b^
Łaziska/Lublin Voivodship/Poland	Mn ^a^		K ^a^	-	Fe ^b^, Zn ^a^, Cu ^a^, Cr ^b^	Fe ^b^, Zn ^a^, Cu ^b^, Cr ^a^
Finland		Na ^a^, Fe ^b^, Zn ^b^, Cu ^b^	Ca ^b^, Fe ^b^, Zn ^b^, Cu ^b^, Cr ^a^	Fe ^b^, Zn ^a^, Cu ^a^, Cr ^b^	-	
Sosnówka/Lublin Voivodship/Poland	Na ^a^	Na ^b^, Fe ^b^, Zn ^b^, Cu ^b^	Ca ^b^, Fe ^b^, Zn ^b^, Cu ^b^, Pb ^b^	Fe ^a,b^, Zn ^a^, Cu ^b^, Cr ^a^		-

^a^ *p* < 0.05; ^b^ *p* < 0.01.

**Table 7:** Results of the Dunn’s test conducted for the analysed data matrix concerning raspberry species cultivated in Poland (*p* < 0.05, *p* < 0.01, and *p* < 0.001).

	*R. idaeus*	*R. occidentalis*/ *R. idaeus*	*R. occidentalis*	*R. idaeus*/ *R. occidenatlis*	*R. chamaemorus*
*R. idaeus*	-	Ca ^a^, Mn ^b^	K ^b^, Na ^c^, Ca ^c^,Mn ^c^, Cu ^a^	Ca ^b^, Mn ^c^	Fe ^a^
*R. occidentalis*/ *R. idaeus*	Ca ^a^, Mn ^b^	-			Ca^b^, Zn^b^
*R. occidentalis*	K ^b^, Na ^c^, Ca ^c^, Mn ^c^, Cu ^a^		-		Na ^c^, Ca ^b^, Zn ^a^, Cu ^b^
*R. idaeus*/ *R. occidenatlis*	Ca ^b^, Mn ^c^			-	Ca ^b^, Cu ^a^, Pb ^a^
*R. chamaemorus*	Fe ^a^	Ca ^b^, Zn ^b^	Na ^c^, Ca ^b^, Zn ^a^, Cu ^b^	Ca ^b^, Cu ^a^, Pb ^a^	-

^a^ *p* < 0.05; ^b^ *p* < 0.01; ^c^ *p* < 0.001.

**Table 8:** Results of the Dunn’s test conducted for the analysed data matrix concerning raspberries of Polish origin (*p* < 0.05, *p* < 0.01, and *p* < 0.001).

	Brzezna/Lesser/Poland	Nałęczów/Lublin Voivodship/Poland	Łaziska/Lublin Voivodship/Poland	Sosnówka/Lublin Voivodship/Poland
Brzezna/Lesser Poland	-	Cu ^a^, Mn ^a^	K ^a^, Fe ^a^, Cu ^a^, Mn ^a^	Na ^a^, Ca ^a^
Nałęczów/Lublin Voivodship/Poland	Cu ^a^, Mn ^a^	-		Na ^b^, Fe ^b^, Zn ^c^, Cu ^c^
Łaziska/Lublin Voivodship/Poland	K ^a^, Fe ^a^, Cu ^a^, Mn ^a^		-	Mg ^a^, Ca ^a^, Fe ^c^, Zn ^b^, Cu ^c^, Mn ^a^, Cr ^a^
Sosnówka/Lublin Voivodship/Poland	Na ^a^, Ca ^a^	Na ^a,b^, Fe ^b^, Zn ^c^, Cu ^c^	Mg ^a^, Ca ^a^, Fe ^c^, Zn ^b^, Cu ^c^, Mn ^a^, Cr ^a^	-

^a^ *p* < 0.05; ^b^ *p* < 0.01; ^c^ *p* < 0.001.

## Data Availability

Not applicable.
